# Long-Read Sequencing Reveals RNA Splicing Complexity in Human Diseases

**DOI:** 10.34133/csbj.0052

**Published:** 2026-04-20

**Authors:** Xiangmin Tan, Ping Wang, Yang Li, Bo Yuan, Qiang Sun, Yaran Liu

**Affiliations:** ^1^ Shandong Key Lab of Complex Medical Intelligence and Aging, Shandong Medical and Pharmaceutical University, Yantai 264003, Shandong, P. R. China.; ^2^ Center for RNA Medicine, the Fourth Affiliated Hospital of School of Medicine, International School of Medicine, Zhejiang University, Yiwu 322000, Zhejiang, P. R. China.; ^3^ Institute of Artificial Intelligence, Beihang University, Beijing 100191, P. R. China.; ^4^ Beijing Advanced Innovation Center for Future Blockchain and Privacy Computing, Beihang University, Beijing 100191, P. R. China.

## Abstract

Transcriptome sequencing is essential for understanding gene expression and RNA features. However, short-read RNA sequencing struggles to analyze complex and full-length messenger RNA molecules. These limitations primarily arise from fragmented read lengths, which make it difficult to accurately characterize alternative splicing patterns, exon structures, or transcription start and termination sites. Long-read RNA sequencing (lrRNA-seq) is an innovative technology that has revolutionized transcriptomic analysis. By end-to-end sequencing, it provides comprehensive insights into transcriptomic structural and regulatory complexity. Moreover, by eliminating the need for transcript assembly and reducing inference errors associated with short-read data, lrRNA-seq can precisely determine exon–intron structures, alternative splicing patterns, transcription initiation and termination sites, alternative polyadenylation, and noncanonical RNA processing events. In this review, we provide a detailed overview of the working principles and technological innovations of lrRNA-seq and emphasize its advantages in transcriptome research. In addition, we systematically assess the methodological aspects, focusing on isoform analysis, quantification, error correction, and algorithm development, which improve the reliability of lrRNA-seq analyses. We further discuss recent applications and developments of lrRNA-seq related to various diseases. Recent studies have revealed disease-related splicing dysregulation, discovered novel pathogenic isoforms, and clarified RNA-mediated mechanisms. Furthermore, we discuss emerging efforts to integrate long-read sequencing with single-cell and spatial transcriptomics, thereby permitting the characterization of splicing complexity across specific cells, tissues, and microenvironments within the whole organism. In conclusion, lrRNA-seq is a transformative technology for advancing disease diagnostics and precision medicine.

## Graphical Abstract

**Figure:**
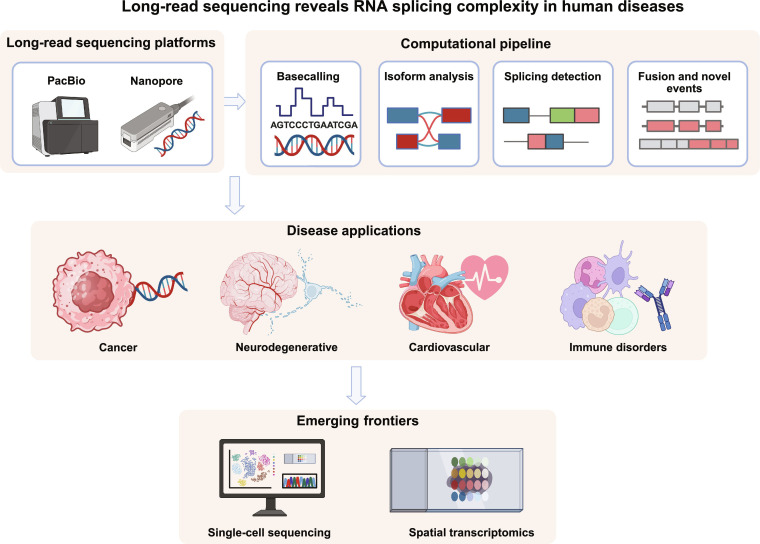

## Introduction

RNA splicing is a process that produces diverse transcript isoforms participating in multiple developmental, physiological, and pathological processes. As an important mechanism that expands transcriptomic and proteomic complexity, it reshapes cellular phenotypes through the selective inclusion or exclusion of exons and regulatory elements [1]. Therefore, characterizing splicing diversity is crucial for deciphering disease mechanisms, cell type-specific regulatory programs, and therapeutic vulnerabilities [2]. Short-read RNA sequencing (srRNA-seq) has long been a primary technology for investigating splicing landscapes due to its technical robustness, reproducibility, and cross-platform consistency [3]. Numerous benchmarking studies have reported high intra- and interplatform concordance [3,4]. Despite these advantages, inherent limitations constrain its ability to resolve transcriptome complexity [5]. A critical step in srRNA-seq library preparation is the fragmentation of messenger RNA (mRNA), which disrupts mRNA integrity and impedes the direct reconstruction of full-length isoforms [6]. Furthermore, ambiguities caused by limited read lengths (50 to 200 bp) make transcript assembly more difficult, particularly for genes with overlapping exon structures [7,8]. Moreover, srRNA-seq is less sensitive for detecting transcripts with low expression levels or those derived from unannotated genomic loci, owing to insufficient coverage and complex reconstruction processes [9]. Consequently, it is particularly challenging to investigate biologically and clinically relevant transcript variants. These limitations emphasize the utility of emerging long-read sequencing methods for accurately capturing full-length isoforms and resolving transcriptomic diversity.

**Fig. 1.**
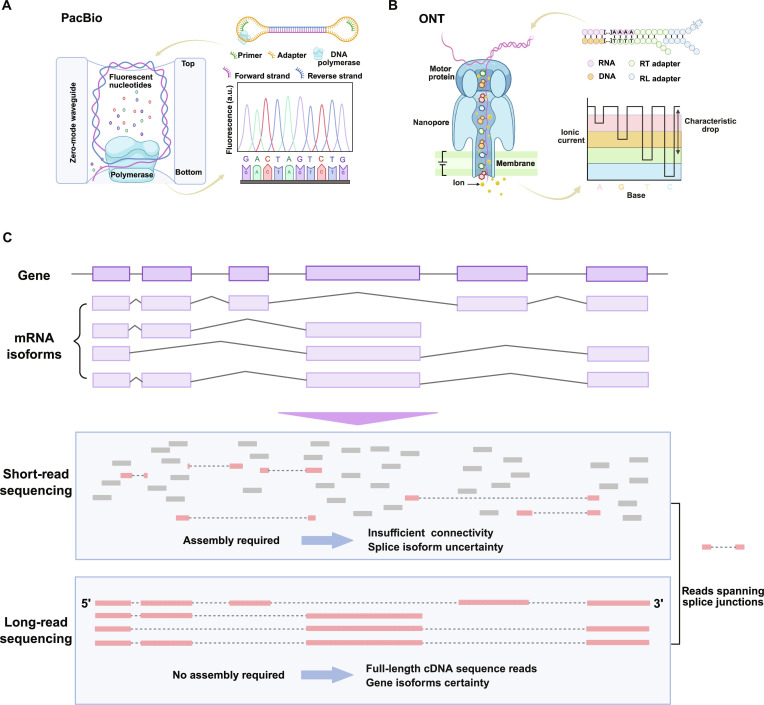
Principles of long-read sequencing technologies and their advantages in transcriptome profiling. (A) Schematic overview of Pacific Biosciences (PacBio) sequencing. DNA or cDNA molecules are circularized with hairpin adapters to form SMRTbell templates and sequenced by a DNA polymerase immobilized within zero-mode waveguides (ZMWs). Real-time detection of fluorescently labeled nucleotide incorporations enables generation of long, high-fidelity reads that can span full-length transcripts. (B) Schematic overview of Oxford Nanopore Technologies (ONT) sequencing. Nucleic acid molecules are translocated through a biological nanopore by a motor protein, and nucleotide sequences are inferred from characteristic disruptions in ionic current. This approach enables real-time sequencing of long DNA or native RNA molecules without amplification. (C) Comparison of short-read and long-read sequencing approaches for transcriptome analysis. Short-read sequencing produces fragmented reads that frequently map ambiguously to exons or splice junctions, leading to splice isoform uncertainty. In contrast, long-read sequencing captures intact transcripts, enabling unambiguous resolution of exon connectivity, alternative splicing events, and transcript isoforms, and providing a more comprehensive view of transcriptomic complexity.

In contrast, long-read RNA sequencing (lrRNA-seq) captures mRNA molecules in a single continuous read, thus facilitating more precise investigation of transcriptome complexity [10–12]. Two main long-read platforms, Pacific Biosciences (PacBio) (Fig. 1A) and Oxford Nanopore Technologies (ONT) (Fig. 1B), routinely generate reads ranging from several kilobases to megabases [13]. They support continuous sequencing of transcripts with distant exons, repetitive elements, and complex structures that cannot be effectively inferred through computational assembly alone [1]. This single-molecule resolution enables simultaneous detection of transcript features, including alternative exon usage, variable transcription start sites (TSSs), and polyadenylation sites (PASs) [1]. Additionally, lrRNA-seq eliminates inherent mapping ambiguities associated with short-read alignments (Fig. 1C), providing more accurate transcript identification and quantification [1]. It has been widely applied to various aspects of transcriptomics, such as the discovery and quantification of full-length transcripts [11,14,15], estimation of poly(A) tail lengths [16,17], and comprehensive profiling of RNA processing events, which focuses on alternative splicing [18,19], back-splicing [20], and alternative polyadenylation (APA) [21]. Altogether, these capabilities make lrRNA-seq an essential technology for studying RNA biology, helping to decipher transcript variations, regulatory mechanisms, and RNA processing.

Providing a comprehensive overview, we detail the core principles of lrRNA-seq and trace its technological evolution, with a particular focus on continuous improvements in read length, accuracy, and throughput. We emphasize the advantages of full-length transcriptome sequencing over conventional short-read approaches. It provides access to transcript structures that cannot be accurately reconstructed from short reads, enabling a more comprehensive view of transcriptome organization. We next discuss how lrRNA-seq can resolve splicing complexity, detailing workflows for basecalling, quality control, read alignment, and isoform-level analysis. These developments support more accurate transcript identification, quantification, and annotation. We next highlight its ability to resolve RNA splicing complexity in various human diseases. Recent studies leveraging lrRNA-seq have revealed previously uncharacterized splicing patterns, differential isoform usage, and coordinated splicing programs, offering new insights into disease mechanisms. Furthermore, the technology is now expanding into single-cell, spatial, and subcellular contexts, allowing the integrated analysis of isoform diversity, cellular heterogeneity, and tissue architecture. Together, these developments represent a crucial shift toward a multilayered understanding of gene regulation in both physiological and disease states.

## Long-Read Sequencing Technology

### PacBio sequencing: Principles, read length, accuracy, and throughput

PacBio sequencing is based on the real-time observation of DNA synthesis at the single-molecule level (Fig. 1A). A circular template (SMRTbell) is generated by ligating hairpin adapters to both ends of a double-stranded DNA molecule [22]. These templates are loaded into nanophotonic wells known as zero-mode waveguides (ZMWs), where individual DNA polymerase molecules are immobilized. As fluorescently labeled nucleotides are incorporated during DNA synthesis, characteristic light pulses are emitted and recorded, enabling real-time base identification [23]. Signals generated across thousands of ZMWs are captured as continuous fluorescence traces and subsequently decoded into continuous long reads (CLRs) [24]. Importantly, the circular structure of the SMRTbell template allows multiple passes of the polymerase over the insert, producing multiple subreads that can be integrated to generate highly accurate consensus sequences.

A key advantage of PacBio sequencing is its long read length, which facilitates comprehensive coverage of complex genomic and transcriptomic regions. Early PacBio RS platforms using C1 chemistry produce reads with an average length of approximately 1.5 kb [25], whereas the PacBio RS II with C4 chemistry achieves mean read lengths exceeding 10 kb [24]. Under optimized conditions, read lengths can extend beyond 60 kb, reflecting the platform’s capacity to span full-length transcripts and structurally complex regions. In contrast, short-read platforms such as the Illumina NovaSeq 6000 typically generate paired-end reads of up to 250 bp, which are often insufficient to resolve repetitive elements and complex splicing events [24].

Initial PacBio sequencing platforms exhibit relatively high error rates (~13%, corresponding to < Q10 accuracy) [26]. However, the development of circular consensus sequencing (CCS) has substantially improved accuracy. In CCS mode, multiple subreads derived from the same SMRTbell molecule are combined to generate a consensus sequence, effectively correcting stochastic errors. With sufficient coverage (e.g., >15 passes), consensus accuracy can exceed 99% [23]. Continued improvements in sequencing chemistry and instrument design have further enhanced performance, with the PacBio Revio platform achieving accuracies of up to 99.95% [27]. In parallel, advances in computational methods, such as DeepConsensus, have further improved basecalling accuracy, enabling HiFi reads to reach Q40 (~99.99%) by correcting systematic insertion and deletion errors [28].

Sequencing throughput has also increased substantially across successive PacBio platforms. The RS II system generates approximately 0.5 to 1 Gb of data per SMRT Cell using 35,000 to 70,000 productive ZMWs [24]. The Sequel platform, with approximately one million ZMWs, increases output to 5 to 10 Gb per run [24,29]. Subsequent improvements in the Sequel IIe enabled the generation of millions of HiFi reads per run, representing a substantial increase in throughput [27]. The latest PacBio Revio system, leveraging high-density flow-cell technology, can achieve outputs of up to ~360 Gb per day [27]. Collectively, these advances have transformed PacBio sequencing from a relatively low-throughput technology into a high-capacity platform suitable for large-scale transcriptomic and isoform-resolved analyses.

### ONT sequencing: Principles, read length, accuracy, and throughput

ONT sequencing is based on the controlled translocation of nucleic acid molecules through a nanoscale protein pore embedded in an electrically resistant membrane. As nucleic acids pass through the pore, characteristic disruptions in ionic current are generated and recorded in real time, enabling the inference of nucleotide sequences [30]. In a typical workflow, cDNA or native RNA molecules are ligated to adapters preloaded with a motor protein that regulates translocation speed through the nanopore [1,30] (Fig. 1B). A tether sequence attached to the adapters localizes the complex to the flow cell surface, enabling pore entry [1]. During sequencing, the motor protein unwinds and translocates nucleic acid molecules through the pore in a stepwise manner, while changes in ionic current are continuously monitored for base identification [1]. Raw signal data are stored in FAST5 or POD5 formats and subsequently processed by computational algorithms for basecalling and sequence reconstruction [1]. Notably, ONT sequencing enables direct analysis of native RNA molecules, allowing simultaneous characterization of nucleotide sequences and RNA modifications.

One hallmark of ONT sequencing is the absence of a built-in limit to read length. Unlike sequencing-by-synthesis technologies, ONT directly measures intact nucleic acid molecules as they pass through the pore, such that read length is primarily determined by the integrity of the input material rather than enzymatic constraints [31]. Advances in DNA extraction and library preparation have therefore substantially extended achievable read lengths [31]. Early Oxford Nanopore MinION runs generate reads on the kilobase scale, whereas optimized protocols now routinely achieve mean read lengths of approximately 20 to 30 kb [32]. These extended read lengths are particularly advantageous for resolving repetitive genomic regions and complex structural features, as demonstrated by the successful assembly of large genomes such as the ~43-Gb lungfish genome [33].

Sequencing accuracy has improved substantially across successive ONT chemistry and software developments. Early R7 nanopores exhibit raw read accuracies of approximately 60% to 70%, which have increased to ~85% to 90% with the introduction of R9 nanopores incorporating engineered CsgG proteins [31]. Concurrent improvements in basecalling algorithms have further enhanced performance, transitioning from hidden Markov models (HMMs) to neural network-based approaches, including Nanocall [34] and DeepNano [35]. Subsequent developments, such as R9.4 and later chemistries, have combined optimized nanopore structures with improved motor enzymes to further increase accuracy [31]. Library preparation strategies have also contributed to accuracy improvements: 2D consensus reads generated with R9.4 chemistry achieve ~92% accuracy, while the R9.5 1D^2^ approach demonstrates accuracies of up to ~95% [36]. More recently, R10 and R10.3 nanopores, featuring dual sensing regions, have improved homopolymer resolution and overall basecalling accuracy [31]. When combined with strategies such as unique molecular identifier (UMI)-based sequencing, accuracies approaching ~99.9% or higher can be achieved, albeit often with reduced throughput [37].

Throughput has also increased markedly with platform evolution. Early Oxford Nanopore MinION devices generate yields on the order of hundreds of megabases per run, whereas current flow cells routinely produce approximately 10 to 15 Gb [31]. Larger-scale platforms, such as Oxford Nanopore PromethION, have substantially expanded sequencing capacity. Individual PromethION flow cells can generate over 100 Gb of data, and the PromethION48 system, operating up to 48 flow cells in parallel, enables total outputs in the terabase range [30]. These advances have established ONT sequencing as a scalable platform suitable for large-genome sequencing, population-scale studies, and comprehensive transcriptomic analyses.

## Dissecting RNA Splicing Landscapes with Long-Read Sequencing

### RNA splicing event types and mechanisms

Eukaryotic cells possess a highly conserved splicing process in which the spliceosome removes introns from pre-mRNA to produce mature mRNA. This controlled RNA splicing enables one gene locus to produce multiple mRNA isoforms, thereby increasing proteome complexity and enabling fine-tuned control of gene function. RNA splicing is tightly regulated by the core spliceosome, cis-regulatory sequences, and trans-acting factors [38]. Among these, trans-acting factors play a key role by modulating the binding of regulatory elements to exons, ensuring accurate splice site selection [38]. Disruption of this regulatory network can trigger aberrant splicing, such as exon skipping, intron retention, or the activation of cryptic splice sites [2]. Driven by mutations in spliceosome components or cis-regulatory elements, splicing dysregulation is increasingly recognized as a key contributor to disease pathogenesis [39]. Thus, understanding gene regulation requires characterizing transcriptomic landscapes and deciphering splicing mechanisms in both normal and diseased conditions.

#### Alternative splicing

**Fig. 2.**
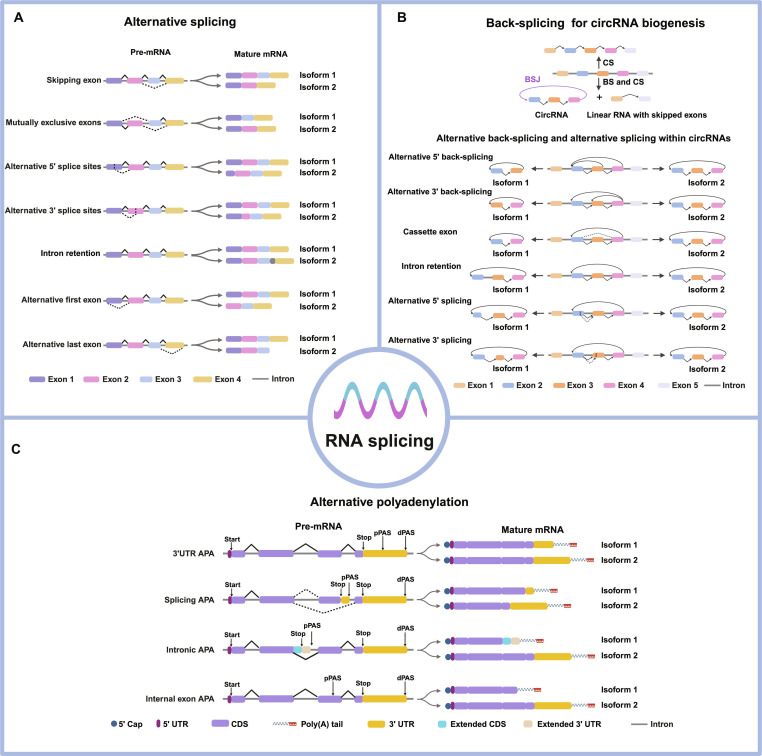
Major mechanisms generating transcript isoform diversity. (A) Alternative splicing of precursor mRNA generates multiple linear transcript isoforms from a single gene through regulated exon skipping, mutually exclusive exons, alternative 5′ and 3′ splice site selection, intron retention, and alternative first or last exon usage, thus diversifying coding potential and regulatory features. (B) Back-splicing-mediated circular RNA (circRNA) biogenesis. In back-splicing, a downstream 5′ splice site is covalently joined to an upstream 3′ splice site, forming a circular RNA molecule. CircRNAs can further undergo alternative back-splicing and internal splicing, giving rise to multiple circRNA isoforms. BSJ, back-splicing junction; CS, canonical splicing; BS, back-splicing. (C) Alternative polyadenylation (APA) generates transcript isoforms with distinct 3′ ends through selective usage of pPAS or dPAS located in terminal exons, introns, or internal exons. APA results in variable 3′ untranslated region lengths or truncated coding sequences, thereby influencing mRNA stability, subcellular localization, and translational efficiency. pPAS, proximal poly(A) site; dPAS, distal poly(A) site.

Alternative splicing is a nuclear regulatory process that generates multiple transcript isoforms through different exon combination patterns. This process is highly common in higher eukaryotes, occurring in more than 95% of multiexon genes [40,41]. Several major alternative splicing patterns have been characterized, including skipping exon, mutually exclusive exons, alternative 5′ splice site, alternative 3′ splice site, intron retention, alternative first exons, and alternative last exons (Fig. 2A) [42]. Among these, skipping exon is the most frequent splicing event in mammalian transcriptomes [43]. Alternative splicing leads to diverse structural and functional protein isoforms, regulating subcellular localization, transcript stability, and translational efficiency [5]. Recent single-cell analyses have shown that alternative splicing is highly variable during early mouse embryonic development [44]. It also exerts broad effects on cell proliferation, apoptosis, metabolism, and differentiation [2]. Its dysregulation is widely linked to human diseases, particularly cancer, where aberrant isoforms disrupt cellular homeostasis and normal gene regulation [45]. Beyond independent functions, crosstalk exists between different layers of RNA regulation [46]. For instance, RNA editing can directly influence the splicing landscape by altering splice site strength when occurring within the splice site sequences [46]. Furthermore, RNA editing can exert indirect control by modulating the binding sites of RNA-binding proteins (RBPs), such as serine/arginine-rich splicing factor 1 (SRSF1), SRSF9, hnRNPA1, and FXR1, thereby affecting their capacity to regulate splicing [46].

#### Alternative polyadenylation

The mRNA maturation entails cleavage of the nascent transcript at its 3′ end, followed by the addition of a poly(A) tail. This essential process contributes to stabilizing mRNA molecules against degradation [45]. The PAS refers to a specific sequence found in 3′ untranslated regions (3′ UTRs), introns, or internal exons. Poly(A) tail addition is not a random event; it occurs with high precision due to PAS recognition [47]. Most eukaryotic genes contain multiple PASs [48]. Variation in PAS usage can result in transcript isoforms with different coding regions or 3′ UTR lengths, a mechanism known as APA [48].

APA occurs in a large proportion of mammalian genes (>70%) [49]. Based on genomic PAS distribution, APA can be divided into 2 distinct types: tandem 3′ UTR APA and upstream APA (including splicing APA, intronic APA, and internal exon APA) (Fig. 2C) [48]. Tandem 3′ UTR APA generates isoforms with variable 3′ UTR lengths, which influence mRNA stability [50], translational efficiency, nuclear export, and subcellular localization [48,51]. Splicing APA produces transcripts with distinctive 3′ UTR sequences, potentially encoding proteins with altered C-terminal domains [48]. Internal exon APA often results in truncated protein products [48]. Thus, APA is critical for governing gene expression and mRNA turnover in various biological processes. Dysregulation of APA has also been widely implicated in human disease. Aberrant PAS usage can impair tumor suppressor function or promote oncogene activation, thereby contributing to disease initiation and progression [52,53].

#### Back-splicing

Circular RNAs (circRNAs) constitute a distinct class of transcripts generated through noncanonical splicing events. The majority are derived from back-splicing, which occurs both cotranscriptionally and posttranscriptionally [54]. During back-splicing, a downstream 5′ splice donor is ligated to an upstream 3′ splice acceptor via a 3′–5′ phosphodiester bond [54]. This process produces a back-splicing junction (BSJ) and yields 2 distinct products: a circRNA and a corresponding linear RNA from which the participating exons are excluded (Fig. 2B) [54]. Alternative back-splicing, which involves the selective use of different 5′ splice donors or 3′ splice acceptors, enables the generation of multiple circRNA isoforms [55]. It is worth noting that circRNAs with multiple exons may undergo further alternative splicing, producing variants that share the same back-splicing site but differ in internal structure [56]. To date, 4 common alternative splicing subtypes have been identified within circRNAs: cassette exons, intron retention, alternative 5′ splicing, and alternative 3′ splicing [56]. For instance, the *CAMSAP1* gene encodes 2 dominant circRNAs differing in intron retention, while the circRNA of the human gene *XPO1* contains a cassette exon [57].

CircRNA expression levels are lower than those of linear forms, mainly due to the inefficiency of the back-splicing process [58]. However, high circRNA expression has been observed, especially in nondividing or slowly proliferating cells, suggesting their regulatory potential [59]. Owing to their closed-loop structure, circRNAs exhibit longer half-lives than linear RNAs and are resistant to exonuclease degradation [60]. They also display distinct subcellular localization relevant to their functions: exon-derived circRNAs typically localize to the cytoplasm [61], while intron-containing circRNAs are often enriched in the nucleus [62]. CircRNAs are engaged in diverse biological processes, regulating gene expression at transcriptional, splicing, and translational levels [59]. They serve as molecular sponges or scaffolds for microRNAs and RBPs [54]. In glioma cells, most circRNAs interact with multiple microRNAs and are linked to cancer-related pathways [63]. Recent studies also implicate circRNAs in disease pathogenesis, with aberrant expression associated with cell proliferation and immunodeficiency [59]. In gastric cancer, dysregulated circRNAs are primarily associated with transcriptional regulation and small GTPase-mediated signal transduction [64]. Similarly, ovarian cancer studies have reported widespread circRNA dysregulation linked to ErbB and mitogen-activated protein kinase signaling pathways [65]. Their abundance, stability, and regulatory capacity position circRNAs as important gene expression regulators and promising biomarkers for diagnosis and therapeutic intervention.

### Benchmark comparison of long- and short-read sequencing in transcriptomic analysis

**Fig. 3.**
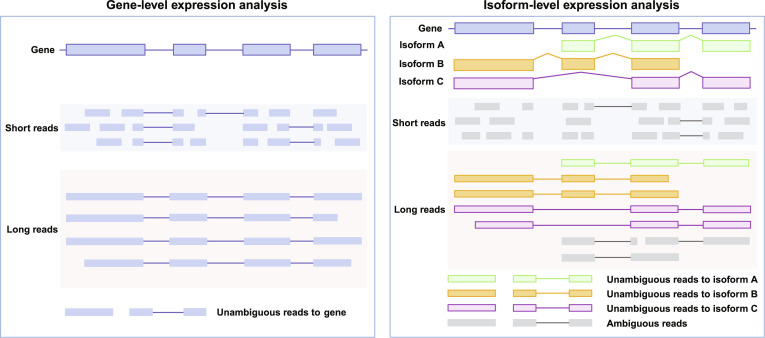
Comparison of gene-level and isoform-level expression analysis using srRNA-seq and lrRNA-seq. The left panel illustrates gene-level expression analysis. Despite differences in read length, both short-read and long-read sequencing allow reads to be assigned unambiguously to genes, resulting in comparable gene-level expression estimates. Short reads represent fragmented transcript segments, whereas long reads often span multiple exons or full-length transcripts. The right panel shows isoform-level expression analysis for a gene that produces multiple transcript isoforms (A to C). Short reads often map to shared exons and splice junctions, resulting in ambiguous read assignment among isoforms and complicating accurate isoform quantification. Long reads, however, frequently span full-length transcripts or large exon combinations, allowing reads to be uniquely assigned to specific isoforms. This capability enables precise identification and quantification of transcript isoforms, highlighting the advantage of long-read sequencing in resolving transcriptomic complexity.

To evaluate the reliability of long-read transcriptome profiling, several studies have benchmarked lrRNA-seq against srRNA-seq data, with particular emphasis on their ability to resolve RNA splicing complexity. Long-read platforms, including Nanopore PCR-cDNA and PacBio Iso-Seq, generally provide more uniform transcript coverage, thereby alleviating the reduced representation at transcript ends that is commonly observed in short-read data due to RNA fragmentation [66]. A key distinction between the 2 technologies lies in their capacity to resolve splicing events. Short-read sequencing infers splicing patterns indirectly from exon–exon junction reads, which often lack sufficient contextual information to reconstruct full-length isoforms, particularly in genes with complex exon organization or repetitive sequences [67]. In contrast, long-read sequencing directly captures full-length transcripts, enabling the simultaneous observation of multiple splice junctions within a single molecule and providing a more accurate representation of exon connectivity and isoform structure [67] (Fig. 3). Regarding read assignment, short-read data typically contain a higher proportion of multimapped reads, which can introduce ambiguity in splice site annotation and lead to misclassification of alternative splicing events, such as exon skipping or alternative splice site usage [66]. Long-read data, by spanning entire transcripts, yield a higher proportion of full-length splice-matching reads that can be more reliably assigned to their source isoforms [66]. Short-read assembly provides high base accuracy but typically generates fragmented transcript reconstructions, limiting its ability to resolve complex splicing patterns across distant exons [68]. Conversely, lrRNA-seq produces more continuous transcript sequences, allowing direct identification of coordinated splicing events, alternative exon combinations, and complex isoform structures, albeit with higher raw error rates [68].

In a head-to-head comparison, Wissel et al. [69] demonstrate that the PacBio Kinnex platform outperforms Illumina in the accuracy of novel transcript discovery, particularly for genes with high isoform diversity and complex splicing patterns. LrRNA-seq also shows clear advantages in identifying novel splicing events, resolving fusion transcripts, and reconstructing immune receptor repertoires, all of which depend on accurate splice junction resolution [70]. For gene-level expression analysis, lrRNA-seq performs comparably to srRNA-seq [69,70] (Fig. 3). However, at the isoform level, short reads typically span only individual exons or splice junctions and therefore often fail to distinguish between transcript isoforms that share common sequences [71] (Fig. 3). In contrast, lrRNA-seq enables direct quantification of full-length isoforms, providing improved accuracy in isoform-level expression analysis and a more faithful representation of splicing diversity [71]. Collectively, these results highlight that while srRNA-seq remains advantageous for high-throughput gene expression profiling, lrRNA-seq offers substantial benefits for resolving alternative splicing and transcript isoform complexity.

### Advantages of lrRNA-seq in characterizing RNA splicing

Conventional srRNA-seq relies on fragmented reads of 50 to 200 bp, which fail to preserve long-range exon connectivity [7,8]. Thus, ambiguous computational assembly increases the difficulty of inferring isoforms from srRNA-seq data, especially for genes with multiple alternative splicing events [67]. Short-read alignments to shared exons or repetitive regions cause multimapping and inaccurate splicing analysis [67] (Fig. 1C). In contrast, long-read platforms analyze intact transcript molecules. This requires no computational reconstruction and allows direct observation of exon connectivity, splicing junctions, and transcript boundaries [1] (Fig. 1C). A direct comparison has shown that ONT sequencing yields a single continuous *DAN4* transcript in yeast, in contrast to multiple fragmented assemblies from short-read data [72]. Therefore, long-read technology provides an unambiguous view of isoform architecture, avoiding the inference errors present in short-read data.

Beyond resolving individual isoforms, lrRNA-seq uniquely enables the characterization of complex and coordinated splicing programs. These include mutually exclusive exons and long-range coordination between distant splicing events or multiexon skipping patterns [30]. For example, PacBio sequencing of colorectal cancer (CRC) has identified tumor-specific isoforms where distal alternative first exon usage is coupled with downstream intron retention in *ARGHDIA*, while proximal alternative 3′ splice site usage coordinates with upstream intron retention in *TMEM259* [73]. These spatially separated yet co-occurring events cannot be resolved through short-read assembly and are found to be highly enriched in tumor cells, revealing an underappreciated layer of splicing dysregulation in cancer. Similarly, PL-Seq has uncovered coordinated regulation of exon skipping and 3′ UTR length within individual *Khc-73* and *Dscam1* transcripts, demonstrating direct coupling between alternative splicing and polyadenylation [21]. Long-read profiling of human tissues has further revealed spatial coordination between transcription initiation and termination, whereby start exon selection influences end-exon usage, supporting a positional initiation–termination axis that links RNAPII elongation dynamics to isoform specification [74].

Indeed, the increased structural detail provided by long-read sequencing has also driven the identification of numerous novel splicing junctions and isoforms. Large-scale studies in different tissues, including the neocortex [75], frontal cortex [76], heart [77], and multiple cancer types [18,78–82], have shown that many isoforms are absent from current gene annotations. These findings are attributed to the accessibility of long-read technology to repetitive, GC-rich regions and structurally complex loci [1]. Similar investigations across diverse tissues and cell types have discovered unexplored splicing complexity, including thousands of novel isoforms even in well-annotated genomes [75,81–86]. More particularly, long-read analysis has been shown to improve the ability to recognize noncanonical patterns, such as intron retention events, gene fusions, and circRNAs [5]. Furthermore, native poly(A) tail composition and length can be measured with ONT direct RNA sequencing, facilitating the analysis of RNA metabolic regulation [87]. It can also be used to study polyadenylation dynamics, mRNA stability, translation efficiency, and their coordinated effects in splicing regulation and gene expression programs [87].

## Computational Pipelines for Deciphering Transcriptome Complexity with LrRNA-seq

### Overview of data analysis pipelines for lrRNA-seq

**Fig. 4.**
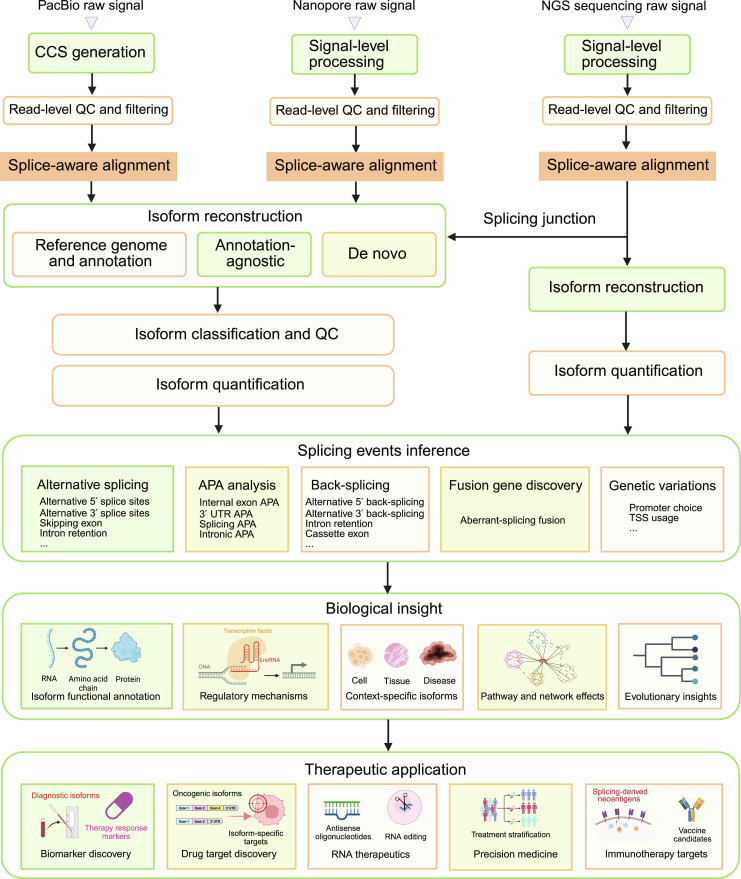
Comparison of transcriptome sequencing data processing workflows for PacBio, ONT, and short-read next-generation sequencing platforms. Raw signals generated by PacBio, ONT, and short-read next-generation sequencing platforms are first converted into nucleotide sequences through platform-specific processing. PacBio data undergo circular consensus sequencing (CCS) generation prior to read-level quality control, whereas ONT and short-read data are processed through signal-level basecalling followed by standard read filtering and quality assessment. Following quality control, reads are aligned to a reference genome or transcriptome. Long-read datasets enable direct isoform identification using reference-guided, annotation-agnostic, or de novo approaches, followed by isoform classification, quality control, and quantification. In contrast, short-read data rely on splice-aware alignment and computational transcript reconstruction based on splice junction inference prior to isoform quantification. The full-length transcript information provided by long-read sequencing facilitates comprehensive isoform-resolved analyses, including alternative splicing, APA, back-splicing events, and fusion transcript detection. These analyses further support downstream functional interpretation, such as isoform annotation, regulatory mechanism exploration, context-specific expression profiling, and pathway-level effects, ultimately enabling applications in biomarker discovery, therapeutic target identification, RNA-based therapeutics, precision medicine, and immunotherapy.

Analysis of lrRNA-seq data involves a multistep computational workflow that transforms raw sequencing signals into biologically interpretable transcriptomic features. Rather than relying on a single analytical strategy, these pipelines integrate multiple stages, including signal processing, quality control, splice-aware alignment, and isoform-level characterization. A key objective of lrRNA-seq analysis is to accurately reconstruct and quantify full-length transcript isoforms while preserving complex splicing patterns. This requires coordinated approaches for identifying splice junctions, resolving exon connectivity, and distinguishing closely related isoforms. Beyond transcript reconstruction, downstream analyses focus on characterizing transcriptome complexity, including alternative splicing events, differential isoform usage, gene fusions, circRNA formation, and functional implications of transcript variation (Fig. 4 and Table 1).

**Table 1.** Comparative summary of bioinformatic analysis methods for srRNA-seq and lrRNA-seq

Analysis phase	Tools	Core algorithm	Input requirements	Strengths	Weaknesses	Suitable application	Ref.
Raw signal processing	BCL-convert v4.4.4	Signal intensity decoding, quality scoring	BCL files	Official Illumina tool; fast demultiplexing	Illumina-only	Illumina	[89]
	Torrent Suite v5.18.1	Signal processing and calibration	Ion Torrent raw signal data	Automated analysis pipeline; rapid data processing	Only for Ion Torrent	Ion Torrent	[88]
	Dorado v5.2.0	Deep learning basecalling model	ONT raw signal data (FAST5/POD5)	High accuracy; efficient	Resource-intensive; ONT-specific	ONT	[90]
	DeepCon- Sensus v1.2.0	HMM, deep learning model	PacBio subreads	mproves HiFi read accuracy and yield	PacBio-only	PacBio	[28]
Read-level QC and filtering	FastQC v0.12.1	Statistical quality profiling	FASTQ files	Comprehensive QC reports; visualization	Not perform trimming	Illumina; Ion Torrent; MGI Tech	[92]
	FASTP v1.2.0	Adaptive quality control with multithreading	FASTQ files	Fast; integrates QC, filtering and trimming	Low parametric flexibility	Illumina; Ion Torrent; MGI Tech	[93]
	NanoPlot v1.46.2	Statistical visualization	FASTQ/BAM files	Comprehensive QC visualization	No filtering functionality	ONT	[95]
	Sequel- Tools v1.0	Rule-based filtering, subsampling	BAM files	Fast and efficient	Limited functionality	PacBio	[94]
	Pychopper v2.7.10	HMMs, dynamic programming algorithm	FASTQ files	Precise identification and orientation	Only for ONT cDNA data	ONT	[96]
Splice-aware alignment	HISAT2 v2.2.1	Graph FM-index, hierarchical indexing	FASTQ files and reference genome	Splice-aware aligner	Less sensitive for complex splicing	Illumina; Ion Torrent; MGI Tech	[98]
	STAR v2.1.3	Seed search, suffix array algorithm	FASTQ files and reference genome	Fast; excellent splice junction detection	High memory requirement	Illumina; Ion Torrent; MGI Tech	[97]
	Minimap2 v2.30	Seed-and-extend, *k-mer* chaining	FASTQ files and reference genome	Fast; widely adopted; supports DNA/RNA	Weaker for complex splicing	ONT; PacBio	[99]
	GraphMap2 v1.0	Graph-based alignment	FASTQ files and reference genome	Robust to high ONT error rates	Slower than Minimap2	ONT; PacBio	[100]
Isoform reconstruction	StringTie2 v3.0.3	Graph-based algorithm	BAM files and reference annotation (optional)	High accuracy and sensitivity	Reference genome-dependent	Illumina; ONT; PacBio	[101]
	Shiba v0.5.1	Statistics framework agnostic to sample sizes	BAM files and reference annotation	Capable of analyzing complex splicing events	External aligner dependency	Illumina; 10x Genomics Chromium	[102]
Isoform reconstruction (annotation-guided)	FLAIR2 v3.0.0	–	BAM files and reference annotation	Strong splice correction; variant-aware	Parameter tuning required	ONT; PacBio	[103]
	Bambu v3.12.1	Machine learning	BAM files and reference annotation	Multisample joint analysis capability	Annotation-dependent	ONT; PacBio	[104]
	IsoTools v2.0.0	Graph-based alternative splicing events identification, β-binomial distribution	BAM files and reference genome sequence and reference annotation	Excellent cross-platform compatibility	Resource-intensive	ONT; PacBio	[106]
	ESPRESSO v1.6.0	EM algorithm	Aligned reads, reference genome and reference annotation	Efficient novel isoform identification	Computationally intensive	ONT; PacBio	[105]
Isoform reconstruction (reference-based but annotation-agnostic)	Mandalorion v4.1.1	Splice-junction-based read clustering, read-supported isoform reconstruction	FASTQ files, reference genome and reference annotation	Superior precision without annotation	Sensitivity to read quality	ONT; PacBio	[108]
	Freddie v1.0	Dynamic programming algorithm, integer linear programming	Aligned reads and reference genome	Annotation-free; high accuracy	Computationally intensive	ONT; PacBio	[107]
Isoform reconstruction (reference-free)	IsoSplitter v1.2	SIM4 algorithms, dynamic programming algorithm, reverse-tracing approach	Aligned reads	Reference-free; high accuracy	Limited AS type detection	ONT; PacBio	[109]
	RNA-Bloom2 v2.0.1	de Bruijn graph	Raw reads (FASTQ)	Reference-free assembly	Computationally intensive	ONT; PacBio; Illumina	[110]
	RATTLE v1.0	Graph-based hierarchical clustering, dynamic programming	Raw reads (FASTQ)	Reference-free; effective error correction	Computationally intensive	ONT	[111]
	isONform v0.3.6	Minimizer pairs, iterative bubble-popping algorithm, SPOA algorithm	Clustered long reads (FASTQ)	Annotation-free; high sensitivity	Requires preprocessing; lower precision	ONT	[112]
Isoform quantification	Salmon v1.11.4	Bayesian inference, EM algorithm	FASTQ files or aligned reads	Fast quantification	Limited discovery in isoform	Illumina	[114]
	IsoQuant v3.12.0	Read counts	FASTQ files or aligned reads	Supports novel isoform discovery	Accuracy depends on alignment quality	ONT; PacBio	[118]
	TranSigner v1.2.0	EM algorithm	FASTQ files	High quantification accuracy	Limited discovery in novel isoform	ONT; PacBio	[115]
	Oarfish v0.9.4	EM algorithm	Raw reads or aligned reads	Superior quantification accuracy	Limited discovery in novel isoform	ONT; PacBio	[116]
	miniQuant v1.4.1	Machine learning, EM algorithm, hybrid SR/LR	FASTQ files, or aligned reads	Better performance for low-expression isoforms	Cost-intensive	ONT; PacBio; Illumina	[117]
Differential isoform expression	DESeq2 v1.0	Negative binomial model	Gene count matrix	Statistically robust	Conservative for small sample sizes	Illumina	[119]
	edgeR v1.0	Empirical Bayes, negative binomial	Gene count matrix	Sensitive for small sample sizes	Weaker outlier handling	Illumina	[120]
	DELongSeq v1.0	Random-effect regression model, EM algorithm	Isoform count matrix	Novel statistical modeling approach	Depends on accurate quantification	ONT; PacBio	[122]
	HBA-DEALS v1.0	Hierarchical Bayesian analysis	Isoform count matrix	Simultaneous modeling of DGE and DAST	Computationally intensive	ONT; PacBio	[123]
Functional impact modeling	NEASE v1.3.2	Network-based enrichment method for AS events	Splicing events	Domain-level resolution	Dependency on annotations	Illumina	[137]
	tappAS v1.1.2	IsoAnnot_Lite_ algorithm	Isoform expression matrix	Functional-level differential analysis	Dependency on functional annotations	ONT; PacBio; Illumina	[127]
	DIGGER v2.0	A collection of standardized algorithms	Isoform ID list and expression data	Isoform-specific interaction analysis	Limited identification in novel interactions	All	[136]
Alternative polyadenylation analysis	DaPars2 v2.1	Piecewise linear regression modeling	Aligned reads and gene annotation	Multisample analysis	Dependence on RNA-seq coverage quality	Illumina	[125]
	DeepPASTA v1.0	Deep learning	RNA sequence data and predicted RNA secondary structures	Detection of complex regulatory signals	Dependence on training data and computational resources	ONT; PacBio; Illumina	[126]
Intron retention detection	IRFinder-S v2.0.1	Convolutional neural network	Aligned reads	Superior IR detection accuracy	Strong input data dependency	Illumina	[124]
circRNA reconstruction and detection	CIRI-full v2.1.1	Forward splice graph (FSG)-based algorithm, Monte Carlo simulation method	FASTQ files	Full-length circRNA reconstruction	Limited by short-read assembly	Illumina	[128]
	CIRI3 v3.0.1	Smith−Waterman algorithm	Aligned reads and reference genome	High sensitivity; BSJ sites detection	Cannot reconstruct full-length circRNA transcripts	Illumina	[129]
	CIRI-long v1.1.0	*k-mer*	FASTQ files and reference genome	High sensitivity; full-length circRNA reconstruction capability	Requirement for high sequencing depth	ONT	[130]
Fusion gene discovery	CTAT-LR-fusion v1.4.0	*k-mer*, de Bruijn graph	FASTQ files and reference genome	Superior detection accuracy	Computationally intensive	ONT/PacBio	[134]
	JAFFAL v2.3	Local re-assembly, precise breakpoint mapping	FASTQ files, reference transcriptome, reference genome, and gene annotation	Precise fusion breakpoint identification	Dependence on annotation	ONT/PacBio	[135]

srRNA-seq, short-read RNA sequencing; lrRNA-seq, long-read RNA sequencing; ONT, Oxford Nanopore Technologies; HMM, hidden Markov model; QC, quality control; circRNA, circular RNA

### Signal-level processing

Raw signal processing is an essential first step in sequencing data analysis, transforming platform-specific signals into nucleotide sequences suitable for downstream analysis. Short-read technologies benefit from relatively simple and stable signal outputs, enabling highly standardized and optimized signal conversion workflows with limited algorithmic variability, as exemplified by established pipelines for fluorescence- and ion-based detection systems [88,89]. In contrast, lrRNA-seq platforms generate more complex and noisy signals, which has led to the development of more sophisticated basecalling strategies, particularly for ONT sequencing. For PacBio sequencing, accuracy is improved by integrating multiple subreads derived from repeated polymerase passes over a circular template into high-fidelity CCS. Early approaches relied on probabilistic models such as HMMs, whereas more recent methods incorporate deep learning architectures to further enhance consensus accuracy, achieving read accuracies exceeding 99% [28], albeit at increased computational cost. Overall, fluorescence pulse-based detection in PacBio systems provides relatively stable base recognition.

ONT sequencing produces ionic current signals characterized by higher variability and noise, which has driven rapid methodological innovation in basecalling algorithms. Recent advances in neural network-based models have substantially improved raw read accuracy and computational efficiency, with current approaches like Dorado v5.2.0 achieving accuracies of up to ~99.75% and enabling hardware acceleration [90]. In addition, these methods support the direct detection of base modifications from native RNA molecules without requiring a reference genome. Despite these advances, challenges remain in accurately interpreting noisy signal patterns, particularly in homopolymer regions and highly modified sequences [91]. Future developments in signal-level processing are expected to focus on improved signal modeling, integration of advanced machine learning frameworks, and enhanced robustness in handling noisy and heterogeneous data, thereby further improving basecalling accuracy and stability.

### Read-level quality control and filtering

Effective quality control and read filtering are essential for obtaining high-quality sequencing data suitable for downstream transcriptomic analysis. SrRNA-seq typically generates reads of 100 to 300 bp, which often exhibit declining base quality toward the read termini. Accordingly, quality control for short-read data primarily focuses on trimming low-quality bases, removing adapter contamination, and handling polymerase chain reaction duplicates through marking or removal. Widely used tools such as FastQC v0.12.1 [92] provide comprehensive summaries of sequencing quality metrics, including per-base quality scores, GC content, and duplication levels, while integrated preprocessing tools such as fastp v1.2.0 [93] enable streamlined workflows for adapter trimming and quality filtering (Table 1). Collectively, these approaches form a standardized framework for short-read quality control prior to downstream analyses.

In contrast, lrRNA-seq data exhibit error profiles that are more evenly distributed along the read length, rendering end trimming less effective. As a result, quality control strategies for long-read data prioritize filtering based on global read quality, length thresholds, and structural completeness rather than base-level trimming. For PacBio data, processing frameworks such as SequelTools v1.0 [94] support rule-based filtering and data subsampling, whereas ONT data are commonly assessed using visualization-oriented tools such as NanoPlot v1.46.2 [95] to evaluate read length distributions and quality metrics. For cDNA-based long-read sequencing, additional processing steps are required to identify full-length transcript molecules and determine read orientation. Adapter-aware approaches, exemplified by tools such as Pychopper v2.7.10 [96], detect and orient full-length reads based on adapter sequences, enabling classification into complete, truncated, or chimeric categories. This step improves transcript boundary definition and reduces artifacts in downstream isoform reconstruction.

### Splice-aware alignment

Splice-aware alignment is a critical step in transcriptome analysis, enabling accurate identification of exon–exon junctions and transcript structures. For srRNA-seq, alignment algorithms typically rely on exact seed-matching strategies. For instance, HISAT2 v2.2.1 [97] employs FM-index-based hierarchical indexing to achieve sensitive spliced alignment with relatively low memory requirements. In contrast, suffix-array-based approaches, exemplified by STAR v2.1.3 [98], identify maximal mappable seeds and generally provide improved alignment performance, particularly for detecting complex or novel splicing patterns, albeit at the cost of higher memory consumption. While these strategies are highly effective for short-read data, their performance declines when applied to long-read sequencing, where reads span multiple splice junctions and exhibit higher error rates.

To address these challenges, long-read alignment methods have been specifically designed to support complex split alignments and tolerate sequencing errors. Minimap2 v2.30 [99], a widely adopted aligner, utilizes a minimizer-based indexing scheme combined with efficient chaining algorithms, achieving a balance between speed and accuracy for lrRNA-seq data. Its scalability and broad applicability have made it a standard choice for long-read transcriptome analysis. However, limitations remain in accurately aligning short exons and resolving highly complex splice junctions. Alternative approaches, such as GraphMap2 v1.0 [100], extend alignment strategies through graph-based models and refined seed-selection mechanisms, improving robustness in repetitive or error-prone regions and enhancing performance for ultralong reads, particularly from ONT platforms. Collectively, these methods highlight the trade-off between alignment speed, accuracy, and sensitivity in detecting complex splicing events. Continued methodological development is required to improve splice junction resolution, particularly in regions with high error rates or intricate exon structures.

### Isoform reconstruction strategies

In srRNA-seq analysis, isoform reconstruction typically depends heavily on reference annotations at the gene or splicing-event level. Methods such as StringTie2 v3.0.3 [101] employ network flow algorithms to assemble and quantify transcripts from spliced alignments, with or without annotation guidance. However, owing to the limited read length and fragmented nature of short-read data, these approaches remain constrained in resolving complex splicing patterns and combinations of multiple alternative splicing events. Although annotation-assisted tools, such as Shiba v0.5.1 [102], can improve the detection of novel splicing events, short-read data often lack sufficient information to reliably reconstruct full-length isoform structures.

In contrast, lrRNA-seq enables individual reads to span entire transcripts, providing a critical advantage for the discovery of novel transcripts. Existing methods can be broadly classified into 3 categories: annotation-guided, reference-based but annotation-agnostic, and de novo approaches. Annotation-guided approaches leverage genome alignments together with existing transcript annotations to infer isoforms through splice-site consistency, intron connectivity, or gene-level modeling. Representative methods, including FLAIR2 v3.0.0 [103], Bambu v3.12.1 [104], ESPRESSO v1.6.0 [105], and IsoTools v2.0.0 [106], incorporate diverse computational frameworks to improve isoform identification and quantification. For example, FLAIR2 v3.0.0 corrects splice junctions using reference annotations and clusters reads based on shared splice junction patterns, while Bambu integrates statistical learning to enable context-aware transcript discovery and quantification. Similarly, ESPRESSO v1.6.0 applies error-aware modeling and gene-level inference to improve splice junction detection and abundance estimation in error-prone long-read data. Collectively, these approaches enhance isoform reconstruction by incorporating prior knowledge to constrain transcript inference. Genome-alignment-based but annotation-independent methods, such as Freddie v1.0 [107] and Mandalorion v4.1.1 [108], aim to identify transcript structures without relying on predefined annotations. These approaches typically partition genomic alignments into exon segments and formulate isoform reconstruction as optimization problems, enabling improved detection of novel and condition-specific splicing events. In addition, fully de novo strategies remove the requirement for a reference genome, facilitating transcriptome reconstruction in nonmodel organisms. Tools such as IsoSplitter v1.2 [109] reconstruct transcript structures by tracing split-read patterns, while methods including RNA-Bloom2 v2.0.1 [110], RATTLE v1.0 [111], and isONform v0.3.6 [112] apply graph-based or clustering-based approaches to assemble and quantify transcripts directly from long-read data. These methods expand isoform discovery beyond annotation-dependent frameworks, although trade-offs between sensitivity and precision may arise depending on data quality and sequencing depth.

Following isoform reconstruction, comprehensive quality control is essential to evaluate transcript model reliability and mitigate biases introduced during sequencing and processing. Tools such as SQANTI3 v5.5.4 [113] assess transcript structures by evaluating TSS, transcription termination sites, and splice junctions, enabling classification and filtering of high-confidence isoforms for downstream analyses. Despite these advances, challenges remain in addressing artifacts arising from RNA degradation, library preparation, and incomplete transcript capture. Continued development of quality control frameworks that integrate bias diagnostics and informative read-level features will be critical for improving the accuracy and interpretability of long-read transcriptome analyses.

### Quantification paradigms

In srRNA-seq analysis, transcript quantification is typically achieved by estimating transcript abundances based on read assignments to reference transcriptomes. Methods such as Salmon v1.11.4 [114] employ selective-alignment strategies to efficiently associate reads with candidate transcripts, followed by probabilistic inference frameworks that account for sequencing biases and fragment distributions. By modeling ambiguity in read assignment, these approaches improve quantification accuracy beyond simple read counting.

In contrast, isoform quantification in lrRNA-seq remains more challenging due to higher sequencing error rates, variability in transcript length, and increased structural complexity of transcriptomes. To address these challenges, current approaches increasingly rely on probabilistic modeling and improved strategies for read assignment. Methods such as TranSigner v1.2.0 [115] and oarfish v0.9.4 [116] utilize expectation–maximization frameworks to assign reads to candidate isoforms, with additional modeling of long-read-specific biases, including coverage heterogeneity. Hybrid approaches, exemplified by miniQuant v1.4.1 [117], integrate both long-read and short-read data to mitigate identifiability limitations in genes with complex isoform structures. In parallel, methods such as IsoQuant v3.12.0 [118] leverage splice-aware alignment information and read clustering to refine isoform-level abundance estimation. As long-read sequencing continues to reveal increasingly complex transcript architectures, future quantification frameworks will likely require tighter integration of transcript structure inference and abundance estimation. Such integration will be essential for achieving accurate and robust isoform-level quantification in complex transcriptomes.

### Downstream inference

#### Differential isoform usage analysis

Differential splicing analysis and isoform expression evaluation aim to reveal changes in transcript usage under condition-specific contexts. Conventional differential expression analysis tools, such as DESeq2 v1.0 [119] and edgeR v1.0 [120], were originally designed for srRNA-seq data and rely on count-based statistical models. Although these tools have also demonstrated utility when applied to long-read data [121], lrRNA-seq presents unique challenges, including transcript selection bias and higher sequencing error rates, which may violate the assumptions underlying traditional count-based frameworks. Therefore, specialized methods for long-read data have been developed. DELongSeq v1.0 [122] extends conventional read-counting strategies by incorporating expression estimates together with their associated uncertainty into a random-effects regression framework, thereby improving the detection of differential isoform expression under the noise characteristics of lrRNA-seq data. In parallel, methods such as HBA-DEALS v1.0 [123] adopt hierarchical Bayesian modeling to jointly infer gene-level expression changes and differential transcript usage, while explicitly accounting for uncertainty in isoform abundance estimation.

#### Specific splicing events analysis

The expanding availability of long-read sequencing enables splicing events to be examined more directly at the isoform level. Tools designed for the detection of canonical splicing events, including exon skipping, alternative 5′ splice sites, alternative 3′ splice sites, and intron retention, generally rely on splice sites and splice junctions as core features for transcript inference. Methods such as FLAIR2 v3.0.0 [103] and ESPRESSO v1.6.0 [105] implement this strategy to reconstruct isoforms and characterize splicing patterns from long-read sequencing data. In addition, specialized approaches have been developed for specific event types; for example, IRFinder-S v2.0.1 [124] focuses on intron retention analysis and employs deep learning to distinguish true retention events from technical noise while supporting multisample and differential analyses.

APA represents another key layer of transcript regulation. In short-read data, methods such as DaPars2 v2.1 [125] infer dynamic APA usage by modeling shifts in PAS usage from read coverage profiles, although such inference is constrained by fragmented transcript information. In contrast, lrRNA-seq enables direct characterization of APA at the isoform level. For instance, DeepPASTA v1.0 [126] applies deep learning to identify polyadenylation signals and predict poly(A) sites from full-length transcript sequences, thereby improving APA resolution. More broadly, integrative frameworks such as tappAS v1.1.2 [127] combine isoform structural features with functional annotations, enabling systematic analysis of alternative splicing, UTR variation, and APA across transcriptomes.

#### CircRNA reconstruction

Reconstruction of circRNAs from srRNA-seq data typically relies on the identification of BSJs, followed by inference of full-length isoforms through assembly. Methods such as CIRI-full v2.1.1 [128] improve isoform-level resolution by integrating splice-site consistency with read coverage, whereas CIRI3 v3.0.1 [129] incorporates enhanced false-positive filtering and graph-based reconstruction strategies to better resolve complex circRNA isoforms, particularly in repetitive genomic regions. These approaches benefit from deep sequencing coverage and achieve high accuracy in BSJ detection. However, the limited read length of short-read data often hinders complete reconstruction of full-length circRNA isoforms and complicates the resolution of structurally complex or repetitive circRNAs. In contrast, long-read sequencing overcomes these limitations by directly capturing full-length transcript sequences. Approaches such as CIRI-long v1.1.0 [130] identify BSJs directly from long reads and reconstruct complete circRNA isoforms, enabling more accurate characterization of circRNA structures and revealing transcript diversity that may be missed by short-read methods.

#### Fusion detection

Gene fusions, whether they arise from cis-splicing, trans-splicing, or basic genomic rearrangements, play a crucial role in tumorigenesis and serve as excellent diagnostic and predictive markers for clinicians [131–133]. Accurate identification of fusion transcripts is crucial for the diagnosis and treatment of diseases such as cancer. LrRNA-seq provides a distinct advantage for fusion detection by directly capturing full-length chimeric transcripts within single reads, thereby overcoming the fragmentation and reconstruction uncertainties inherent to srRNA-seq [134].

Most long-read fusion detection pipelines use alignment tools capable of recognizing splicing events to identify chimeric sequences mapped to multiple genomic loci or genes. CTAT-LR-fusion v1.4.0 [134] integrates evidence from both lrRNA-seq and srRNA-seq data to maximize fusion detection sensitivity across bulk and single-cell datasets. JAFFAL v2.3 [135] develops a heuristic filtering method and combines it with a long-read alignment strategy, effectively overcoming the high sequencing error rate of lrRNA-seq data. By precisely identifying breakpoints relative to exon boundaries, it also reduces chimeric read artifacts introduced during cDNA library preparation. Collectively, these tools further enhance the sensitivity and accuracy of fusion event detection and facilitate subsequent functional and clinical interpretation.

#### Functional impact modeling

Beyond statistical detection of differential isoform expression, functional interpretation frameworks link splicing changes to regulatory mechanisms and biological consequences. For instance, tappAS v1.1.2 [127] introduces the Functional Iso-Transcriptomics framework, which integrates differential isoform usage with functional annotation of protein domains and motifs to assess the regulatory and functional impact of alternative isoform expression. DIGGER v2.0 [136] extends its functional interpretation into systems biology by interweaving protein–protein interaction networks, domain–domain contacts, and residue-level information. It illustrates how variations in specific isoforms or exons can rewire interaction networks and disrupt the architecture of pathways. In parallel, NEASE v1.3.2 [137] focuses on pathway-level interpretation by performing enrichment analyses informed by structural annotations of protein interaction networks using differential splicing events as input. This approach identifies splicing-driven pathway alterations and potential biomarkers, providing a complementary perspective to conventional pathway analysis and facilitating interpretation of tissue-specific and disease-associated splicing regulation.

## LrRNA-seq in Human Diseases: Mechanistic Insights and Therapeutic Implications

As long-read sequencing continues to improve, becoming more versatile, accurate, faster, and affordable, its applications have expanded widely. By resolving transcript architectures at the isoform level, lrRNA-seq uncovers unrecognized splicing alterations that drive disease progression [18,138,139]. Such insights are crucial for clarifying underlying molecular mechanisms and identifying clinically relevant biomarkers.

**Fig. 5.**
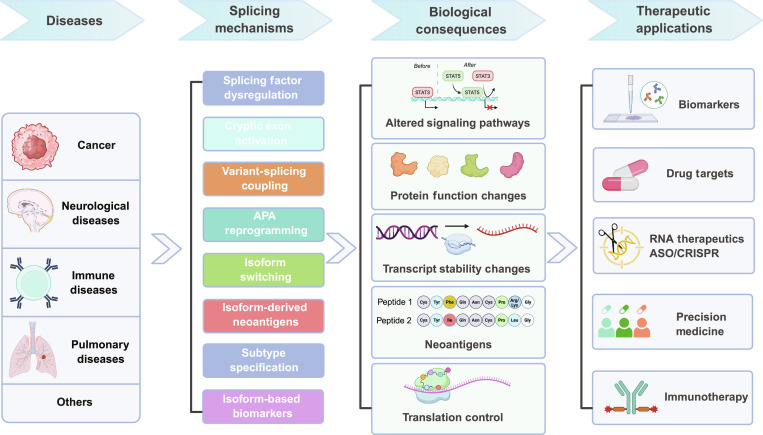
Splicing dysregulation as a central driver of disease mechanisms and therapeutic opportunities. Aberrant RNA splicing represents a fundamental layer of gene regulation that is widely disrupted across diverse human diseases, including cancer, neurological, immune, and pulmonary disorders. Multiple mechanisms contribute to splicing dysregulation, such as alterations in splicing factors, activation of cryptic exons, coupling between genetic variants and splicing, APA reprogramming, and isoform switching. These perturbations reshape transcript isoform landscapes and lead to diverse biological consequences, including altered signaling pathways, changes in protein function, transcript stability, and translational regulation, as well as the generation of isoform-derived neoantigens. Collectively, these molecular alterations drive disease phenotypes and contribute to subtype specification. Importantly, splicing-derived isoforms provide a rich source of clinically actionable targets. Their applications span biomarker discovery, identification of drug targets, development of RNA-based therapeutics (e.g., antisense oligonucleotides and CRISPR-based approaches), patient stratification in precision medicine, and immunotherapy targeting neoantigens.

Different diseases can share the same signaling pathways [140]. The following section will discuss the application of lrRNA-seq across multiple diseases, highlighting the shared mechanisms at the isoform level (Fig. 5). The value of long-read sequencing extends beyond mechanistic discovery to therapy development. Several promising strategies are emerging, including small-molecule inhibitors [141] and targeted approaches to correct aberrant splicing. Notably, CRISPR/dCasRx-mediated splicing regulation [18] and splicing-factor-targeted antisense oligonucleotides (ASOs) [142] have shown promising therapeutic results. Collectively, these results highlight the importance of lrRNA-seq as a tool for understanding disease causes and developing new therapeutic approaches.

### Splicing factor dysregulation drives disease-associated isoforms

Mutations or expression changes in core splicing factors (e.g., SF3B1, SRSF2, and U2AF1) represent important contributors to splicing dysregulation observed in cancer [18,141–145]. Such dysregulation can act as an oncogenic driver by generating aberrant transcript isoforms that contribute to tumorigenesis through diverse molecular mechanisms, including altered signaling pathways and disrupted cellular homeostasis [146]. SF3B1, one of the most frequently mutated RNA splicing factors across cancers, is particularly prevalent in hematological malignancies [145]. In chronic lymphocytic leukemia (CLL), SF3B1 mutations drive the aberrant inclusion of exon 15 in *BRD9*, leading to the generation of a truncated isoform that can stabilize the noncanonical BRG1/BRM-associated factor chromatin-remodeling complex, increase chromatin accessibility, and contribute to leukemic progression [141]. Notably, SF3B1-mutant CLL cells exhibit selective vulnerability to BRD9 inhibition, revealing a therapeutic opportunity directly linked to isoform dysregulation [141]. In addition to mutations, altered expression of splicing regulators can also drive disease-associated isoform changes. Long-read transcriptome profiling has revealed that SRSF1 is up-regulated in CRC [18]. Mechanistically, SRSF1 promotes CRC progression by sustaining the inclusion of exon 4 to 5 in the *TIMP1* gene, as its knockdown attenuates malignant phenotypes, including cell growth, migration, and invasion [18]. Furthermore, dysregulation of epithelial splicing regulatory proteins (ESRP1/2) represents another mechanism of isoform switching. In gallbladder cancer, up-regulation of ESRP1/2 generates a novel isoform (*ERBB2 i14e*), which enhances ERBB2–ERBB3 dimerization and promotes AKT phosphorylation, thereby contributing to tumor proliferation [142]. Collectively, these findings illustrate that splicing factor dysregulation can reshape isoform landscapes and drive disease-associated molecular phenotypes.

### Cryptic exon activation contributes to disease development

In both frontotemporal dementia (FTD) and chronic obstructive pulmonary disease (COPD), activation of cryptic splice sites has emerged as an important molecular mechanism that disrupts gene function. In frontotemporal lobar degeneration with TAR DNA-binding protein 43 (TDP-43) pathology, aberrant phosphorylation and mislocalization of TDP-43 lead to its nuclear depletion and cytoplasmic aggregation, which in turn drives widespread cryptic exon activation and contributes to axonal dysfunction and neurodegeneration [76]. For example, inclusion of cryptic exons in *STMN2* and *ARHGAP32* induces premature polyadenylation, resulting in nonsense-mediated decay (NMD) or the production of truncated proteins [76]. In COPD, activation of a cryptic splice acceptor site in the *NPNT* gene leads to the insertion of an additional serine residue in the encoded protein [147]. This isoform alteration is associated with increased COPD risk and reduced lung function [147]. Collectively, these findings demonstrate that cryptic exon activation represents a conserved mechanism of transcript disruption across diverse diseases, linking splicing dysregulation to functional impairment at the molecular and physiological levels.

### Genetic variant–splicing coupling shapes disease-associated transcript diversity

Genetic variation in the human genome can alter the splicing of disease-associated genes by disrupting regulatory elements within splicing hotspots [148]. The clinical manifestations of many complex diseases often arise from the combined effects of multiple genetic variants [149]. Long-read data have revealed 2 major mechanisms by which genetic variation regulates gene expression: (a) variants influence spliceosome recognition of specific exons, thereby altering protein-coding sequences [85]; and (b) they affect promoter choice or TSS usage, modifying the 5′ end structure of mRNAs and consequently influencing gene expression levels [85,148]. Long-read transcriptome profiling of human brain tissues has identified a broad spectrum of splicing events associated with genetic variants implicated in neurological disorders, including bipolar disorder, schizophrenia, Alzheimer’s disease (AD), Parkinson’s disease (PD), anxiety, and depression [150].

Through colocalization analysis of splicing quantitative trait loci (sQTLs) with genome-wide association studies (GWAS), Humphrey et al. [84] demonstrate that the splicing pattern of *PLCG2* is influenced by the AD risk variant rs12446759. Similarly, exon 4 skipping in *SIPA1L2* has been linked to the sQTL rs16857578 in PD [84]. In the schizophrenia-associated gene *CACNA1C*, a known risk variant located within intron 3 colocalizes with a newly identified alternative splicing hotspot in exon 7, generating multiple novel isoforms [151]. In addition, integrative analyses combining GWAS, sQTL mapping, and targeted lrRNA-seq have shown that a COPD-associated variant (rs34712979) alters splicing of the *NPNT* gene and influences isoform expression and usage in human lung tissue [147]. Collectively, these findings demonstrate that genetic variant–splicing coupling represents a critical mechanism underlying transcriptomic diversity in disease. By linking genetic variation to splicing dysregulation and phenotypic outcomes, lrRNA-seq provides a powerful framework for understanding the molecular basis of complex diseases.

### APA reprogramming reshapes gene expression

A common pathogenic mechanism of APA reprogramming across various diseases involves modulating microRNA binding to influence the stability and expression of target transcripts. Using ONT sequencing, Zhang et al. [152] have demonstrated widespread APA reprogramming in ulcerative colitis (UC) tissues, characterized by remarkable changes in PAS usage and 3′ UTR length. APA site selection influences mRNA stability and translation efficiency within a broader regulatory network, potentially modulating the activity of microRNA and RBPs. Notably, 5 APA-associated isoforms, namely, *CD38*, *NCALD*, *SMIM31*, *GPX7*, and *SWAP70*, are identified as potential molecular drivers of UC pathogenesis [152]. Long-read sequencing of CRC has characterized extensive 3′ UTR shortening across numerous genes resulting from APA reprogramming [144]. These shortened 3′ UTRs contribute to tumor progression by enhancing gene expression and evading microRNA-mediated repression [144]. In breast cancer, an APA isoform of *TLE1* encodes a truncated protein that disrupts the transcriptome in MCF-7 cells and promotes oncogenic phenotypes [153]. Collectively, these findings highlight APA reprogramming as a key regulatory mechanism that reshapes gene expression landscapes and contributes to disease progression through posttranscriptional control.

### Isoform switching as a driver and therapeutic target

Guided by long-read sequencing, isoform-centric analyses have revealed that pathogenic dysregulation in various diseases occurs predominantly through isoform switching rather than changes in total gene expression [154]. In systemic lupus erythematosus, a critical pathogenic event involves an isoform switch in the *IRAK1* gene [154]. Although total *IRAK1* mRNA levels are comparable between patients and healthy controls, the proportion of a functional full-length isoform (ENST00000393687.6) is markedly increased relative to a novel truncated isoform (*IRAK1-1*) [154]. This shift toward the full-length isoform contributes to hyperactivation of the TLR7/9-IRAK1-IRF7 signaling pathway, ultimately leading to excessive type I interferon production [154]. Similarly, in rheumatoid arthritis, a pathogenic isoform switch occurs in the *SIGLEC10* gene, a key negative regulator of inflammation [154]. Although total *SIGLEC10* mRNA levels are elevated in patients, there is a pronounced increase in the proportion of novel isoforms that are substrates for NMD [154]. The consequent predominance of nonfunctional isoforms diminishes the anti-inflammatory activity of SIGLEC10, exacerbating disease pathology [154].

In CRC, a switch from the tumor-suppressive *TIMP1 Δ4-5* isoform to the oncogenic full-length *TIMP1-FL* variant contributes to tumor progression [18]. Importantly, CRISPR/dCasRx-mediated skipping of exon 4 to 5 restores this protective splicing pattern and attenuates tumor growth, highlighting the therapeutic potential of targeted splicing modulation [18]. In COPD, isoform switching in the *NPNT* gene, involving the incorporation of a serine residue near protein N-terminus, directly contributes to disease pathology [147]. In gallbladder cancer, full-length transcriptome profiling has shown that splicing switching of the *ERBB2* gene promotes cell proliferation and confers resistance to trastuzumab [142]. Notably, targeting *ERBB2 i14e* with ASO in combination with trastuzumab or the antibody–drug conjugate T-DXd markedly suppresses tumor growth [142].

### Isoform-driven immune modulation through neoantigen generation

Long-read transcriptomic analyses reveal an additional immunogenic dimension to splicing dysregulation: the generation of unrecognized neoantigens. Presented on human leukocyte antigen (HLA) class I molecules, these peptides can potentially trigger cytotoxic T-cell responses, thereby directly linking splicing alterations to tumor immunogenicity [155]. Long-read sequencing approaches uncover aberrant splicing-derived open reading frames that serve as a rich source of immunogenic peptides [156]. A PacBio-based transcriptomic atlas of CRC demonstrates that tumor-specific isoforms generate 157 neoepitopes from EpiT-specific open reading frames, yielding recurrent peptides optimized for HLA population coverage [73]. In non-small cell lung cancer (NSCLC), MinION sequencing has identified more than 2,000 aberrantly spliced isoforms that can be translated into peptides, and subsequent ELISpot assays demonstrate that selected candidates can activate HLA-restricted T-cell responses [156]. Consistently, another long-read NSCLC study has reported 38,058 novel isoforms, greatly expanding the potential neoantigen repertoire [157].

In uveal melanoma, ONT sequencing has identified 3 isoform-derived peptides that can induce strong T-cell responses and mediate tumor cell killing [158]. In lung adenocarcinoma, retention of intron 12 in *STAT2* functions as a dominant-negative regulator of type I interferon signaling [159]. Other large-scale long-read transcriptomic studies in breast cancer [79], papillary thyroid microcarcinoma (PTMC) [80], and gastric cancer [139] have consistently reported widespread tumor-specific transcripts that serve as upstream sources for neoantigen discovery, even in the absence of direct immunological validation. Together, these studies highlight lrRNA-seq as a powerful tool for uncovering isoform-derived neoantigens, thereby broadening therapeutic opportunities in cancer immunotherapy.

### Isoform profiling enables precise molecular subtype classification

From neural development to malignant tumor, differences at the isoform level, rather than gene-level expression, serve as a precise molecular determinant of cellular identity and clinical subtypes. For example, lrRNA-seq of the developing neocortex has shown that isoform-level expression variation distinguishes cellular identities, reflecting cell-type-specific differences in TSS selection and PAS usage [150]. Exon skipping of *TCF12* occurs more frequently in astrocytes than in neurons under physiological conditions [160]. Isoform-level profiling is increasingly recognized as a critical dimension of precision oncology. Long-read transcriptome analysis of leukemia has uncovered numerous previously unannotated isoforms, the diversity of which defines 8 molecular subtypes with distinct prognostic outcomes and immune landscapes [82]. In addition, long-read sequencing has revealed distinct splicing event profiles that classify acute myeloid leukemia into U2AF1-like and SRSF2-like subtypes [161]. Notably, the U2AF1-like signature is associated with poor survival and enhanced leukemic growth [161]. In PTMC, long-read profiling has identified novel tumor-specific gene isoforms of *FRMD3*, *NOD1*, and *SHROOM4* that are enriched in the pN1b subtype [80]. A long-read sequencing in CRC has revealed that splicing alterations, including 3′ UTR shortening, are preferentially enriched in stem/transit-amplifying-like malignant cells [144] Similarly, a PacBio-based CRC atlas has identified dysregulated transcript architectures that distinguish epithelial subpopulations with distinct prognostic associations [73]. Collectively, these findings demonstrate that isoform-level profiling provides a refined molecular framework for disease classification, enabling more precise stratification of cellular states and clinical subtypes than gene-level analyses.

### Isoform-based biomarkers for disease diagnosis and prognosis

LrRNA-seq has enabled the identification of isoform-level biomarkers with diagnostic and prognostic value across diverse diseases. Using ONT sequencing, Qu et al. [162] characterize the transcriptomic landscape of CRC with mismatch repair deficient and microsatellite instability and have identified *INHBA* as a robust plasma biomarker that may improve patient stratification for targeted therapies. In lung adenocarcinoma, retention of intron 12 in *STAT2* has been proposed as a predictive biomarker for response to programmed cell death-ligand 1 blockade therapy [159]. In breast cancer, an alternative first-exon isoform of *CYB561* and an exon-skipping isoform of *CEACAM1* are associated with poor clinical outcomes [79]. Similarly, in gallbladder cancer, the *ERBB2 i14e* isoform is overexpressed and correlates with reduced patient survival [142]. In gastric cancer, lrRNA-seq has revealed numerous promoter-driven isoforms, including truncated variants of *MET*, *FGFR4*, and *ERBB3*, that are associated with poor prognosis [139]. LrRNA-seq has also facilitated the exploration of mitochondrial RNA splicing in neurodegenerative diseases. For example, Aguzzoli et al. [83] have identified 4 isoforms from the *MT-RNR2* locus, which encodes mitochondrial 16S rRNA and partially translates into an anti-apoptotic peptide, highlighting their potential as biomarkers of mitochondrial dysfunction. Together, these findings highlight the capacity of lrRNA-seq to identify isoform-level biomarkers relevant to disease classification, therapeutic response, and prognosis.

## Emerging Trends and Future Opportunities in Long-Read Sequencing

### Long-read single-cell sequencing

The regulation of RNA splicing is different across tissues, anatomical regions, and cell types, while bulk transcriptomic profiling has an inherent constraint: it averages signals across heterogeneous cell populations, often obscuring isoform- or cell-type-specific expression patterns [163,164]. This loss of resolution can diminish sensitivity for detecting biologically meaningful differences between conditions or disease states [13]. This is especially noticeable in tumor tissues, which comprise complex mixtures of malignant, stromal, immune, and extracellular matrix components, as disease-specific signals can be diluted [13,164]. Single-cell RNA sequencing (scRNA-seq) overcomes this limitation by resolving cellular heterogeneity. Its methodology, based on labeling transcripts with UMIs and cell barcodes [165], enables the high-resolution identification of resistant clones, lineage trajectories, and cell-state-specific responses [13]. Through microfluidic or plate-based capture, individual cells are isolated, lysed, and converted into barcoded cDNA libraries, allowing sequencing reads to be traced back to their original cell [165]. This makes it particularly powerful for profiling tissues with diverse cellular compositions.

However, even high-resolution short-read single-cell sequencing struggles to capture critical structural variants, resulting in incomplete resolution of genomic heterogeneity [164,166]. In contrast, single-cell long-read sequencing directly resolves full-length transcripts within individual cells, eliminating the ambiguities of short-read assembly and enabling precise characterization of splicing patterns, exon connectivity, and transcript boundaries. The resulting isoform-resolved single-cell atlases reveal regulatory features and cellular heterogeneity obscured in bulk or short-read analyses. For example, single-cell long-read studies have uncovered extensive splicing dysregulation across malignant cell populations, including complex combinations of splicing events [73], tumor-specific neoantigens [73], subtype-specific isoform switching [144], and transcript structural alterations driven by single-nucleotide variants [167].

Computational tools have been developed to address the analytical difficulties of error-prone long-read data at the single-cell level. The use of scNanoGPS has enabled deconvolution of scNanoRNA-seq reads for individual cells and molecules, making it possible for simultaneous genotyping and phenotyping [168]. Analysis of kidney tumors has displayed that cell-type-specific splicing variation is ubiquitous and undetectable in conventional gene-level expression analyses [168]. In a study of progranulin gene-deficient FTD using SnISOr-Seq2, about 30% of disease-associated splicing perturbations are specific to particular neuronal or glial cell types [160]. Taken together, these findings show that single-cell, long-read sequencing has provided a paradigm-shifting tool for studying RNA splicing in disease, enabling isoform-level identification of cellular heterogeneity.

### Long-read spatial transcriptomics

Although capable of comprehensive transcriptome profiling, single-cell long-read sequencing destroys cellular structure and fails to capture spatial information. To solve this problem, spatial transcriptomics have been developed to preserve tissue architecture while capturing transcriptomic information in situ. Unlike scRNA-seq, which tags each cell with a barcode, spatial transcriptomics utilizes positional barcodes to map transcripts to their physical locations within a tissue section [169]. Typically, each barcode covers a capture area containing several cells, so the resolution is coarser than the true single-cell level [169]. Subsequent methodological advances have steadily enhanced spatial resolution from near-cellular to subcellular scales [170], establishing a foundation for integrating spatial transcriptomics with long-read sequencing to bring isoform-level resolution to spatially organized biological contexts.

Integration of spatial analysis and lrRNA-seq has already provided insights into complex biological tissues. For example, long-read spatial transcriptome mapping of pediatric and adult gliomas by the 10X Visium platform has decoded niche-specific multicellular ecosystems and identified *FAM20C* as a key factor in tumor infiltration [171]. Similarly, integration of Spl-ISO-Seq with the human visual cortex has provided spatially resolved splicing regulation at a nearly single-cell level, providing new perspectives on brain development and neurological disorders [172]. A frontier in transcriptomics lies in merging lrRNA-seq with spatial technologies to investigate how isoform expression and processing vary across tissue regions, uncovering principles of cellular organization.

## Unresolved Challenges and Open Methodological Questions

### Artifactual transcripts from library preparation and reverse transcription

During library preparation, artifacts originating from the reverse transcription reaction, such as template switching, internal priming, modification-induced errors, mis-priming, and primer-independent priming, can increase uncertainty in long-read transcriptome analyses [173]. Reverse transcription errors, such as mis-priming driven by RNA secondary structure, can generate artifactual cDNA molecules that may be misinterpreted as genuine structural variants or novel isoforms [1,173]. Incomplete coverage of long transcripts resulting from reverse transcriptase nonprocessivity or RNA degradation can negatively affect both mapping accuracy and transcript quantification [1,174,175]. In addition, high template concentration, low temperatures, and homologous repeats act as risk factors that promote template switching, a process that may be further exacerbated during long-read library preparation [176]. The resulting artifacts frequently generate spurious poly(A) sites in short A-stretches lacking polyadenylation signals, complicating accurate annotation of APA [176]. These issues underscore the need for improved library preparation strategies and quality control procedures to minimize artifactual transcript detection.

### Biological versus technical intron retention

Distinguishing genuine biological intron retention from technical artifacts remains a major challenge in lrRNA-seq analyses. Sequencing errors near splice junctions can cause misalignment, resulting in incorrect splice junction predictions [1]. This challenge is further compounded by the inherent characteristics of long-read sequencing data. In addition, mapping ambiguity and incomplete transcript coverage can further confound the identification of true intron retention events [177]. Elevated error rates, particularly insertion–deletion errors, can obscure splice junction boundaries and lead to false-positive intron retention calls [177]. Moreover, partial transcript coverage caused by RNA degradation or incomplete reverse transcription may result in reads that appear to retain intronic sequences, even when the corresponding transcripts are fully spliced [177].

From a biological perspective, intron retention is often tightly regulated and associated with specific cellular states, developmental stages, or stress responses [42]. Genuine intron-retained transcripts frequently exhibit distinct features, such as conserved splice site motifs, regulated expression patterns, and reproducibility across biological replicates [42]. In contrast, technical artifacts are more likely to be stochastic, sample-specific, and unsupported by independent evidence [175]. Consequently, accurately resolving intron retention requires careful integration of sequencing depth, alignment quality, and orthogonal validation strategies to differentiate biological signals from technical noise [177]. Approaches such as cross-platform validation with short-read data, consistency across replicates, and the incorporation of splice-aware error correction can substantially improve the reliability of intron retention detection [177].

### Overestimation of transcript diversity and technical biases

LrRNA-seq exhibits an inherent coverage bias toward highly expressed genes, whereby abundant transcripts consume a disproportionate fraction of sequencing reads, leading to insufficient coverage of low-abundance transcripts [66]. This limitation is further compounded by the relatively low throughput of direct RNA sequencing, which constrains sequencing depth, as well as by higher error rates compared to short-read and PacBio technologies, thereby posing challenges for accurate transcript identification [66,178]. As a result, replicability, particularly for low-abundance transcripts, remains a key concern in lrRNA-seq studies [179]. Although deeper sequencing improves the detection of low-abundance transcripts, it does not consistently improve transcript reconstruction accuracy, as increased depth may also amplify technical noise and spurious transcript models [1,174]. Consequently, distinguishing genuine rare transcripts from technical noise remains a major challenge.

In addition, length-selection bias inherent to protocols such as Iso-Seq and Kinnex introduces confounding signals into splicing event analysis [1]. These biases are further compounded by variability in sequencing platform performance and library quality, which together influence transcript identification and contribute to inconsistencies in transcriptome profiling. Collectively, these technical factors may lead to overestimation of transcript diversity in long-read analyses. To address these limitations, the use of external standards, such as spike-in RNA variants, provides a practical approach for assessing technical noise levels [177]. Nevertheless, current data processing pipelines still require substantial improvement to reliably distinguish genuine transcripts from stochastic noise caused by splicing errors or sequencing artifacts [1,179]. Finally, despite the advantage of longer reads, accurate mapping within repetitive regions larger than 100 kb remains challenging, which further complicates the detection of structural variants in these genomic contexts [180].

### Ground-truth validation of novel isoforms

Given the technical uncertainties associated with lrRNA-seq, systematic strategies are required to validate newly identified transcript isoforms. One commonly adopted approach is cross-platform validation, in which high-coverage srRNA-seq data are aligned to candidate isoforms to assess whether exon–exon junctions detected in long-read datasets are supported by independent short reads spanning the same splice sites [18,73]. In addition to computational support, experimental validation provides critical orthogonal evidence. Targeted reverse transcription-polymerase chain reaction is widely used to confirm predicted exon connectivity and splice junction usage [18]. Furthermore, proteomic evidence obtained through mass spectrometry can help determine whether a transcript isoform is translated into a functional protein product, thereby providing functional validation at the protein level [156].

Beyond individual validation approaches, reproducibility across independent samples represents an important criterion for transcript authenticity. Genuine isoforms are expected to recur across biological replicates or independent cohorts, whereas artifactual transcripts are more likely to be stochastic and sample-specific. Collectively, these complementary strategies provide a framework for establishing ground truth in long-read transcriptomics. However, the lack of standardized validation pipelines and benchmark datasets remains a major limitation, highlighting the need for more systematic and scalable validation frameworks.

### Complementarity of sequencing platforms and hybrid strategies

Despite the advantages of lrRNA-seq in resolving transcript structures and isoform complexity, srRNA-seq remains indispensable for several applications. In particular, for large-scale gene expression studies involving multiple conditions or replicates, short-read platforms provide higher throughput and lower per-sample cost, making them more suitable for analyses requiring strong statistical power [174]. In addition, srRNA-seq generally offers greater sensitivity for detecting low-abundance transcripts and provides more robust quantification [66,179]. However, short-read technologies are limited in reconstructing full-length transcript structures, whereas long-read sequencing enables highly contiguous assemblies but may suffer from reduced base accuracy, particularly at low coverage [68]. These complementary strengths highlight that no single platform is sufficient to fully resolve transcriptome complexity.

Hybrid sequencing strategies that integrate short-read and long-read data therefore represent an effective solution to these trade-offs. By combining the high accuracy and depth of short reads with the structural resolution of long reads, hybrid approaches can improve both transcript identification and quantification. Empirical studies support this strategy. For example, Yorki et al. [68] systematically evaluate hybrid assembly approaches and demonstrate that integrating short-read and long-read data achieves an optimal balance between assembly continuity and accuracy. Consistently, several transcriptome analyses further suggest that hybrid strategies provide a robust framework in which short reads support accurate quantification, error correction, and high-throughput screening, whereas long reads resolve full-length transcript structures and enable functional validation [18,73,76,78,181].

Empirical studies support this strategy. For example, Sun et al. [18] employ ONT sequencing as the primary platform for novel isoform discovery, and use srRNA-seq data for error correction, quality control filtering, and accurate expression quantification with StringTie. Similarly, Faura et al. [76] use the Illumina HiSeq 4000 platform across 149 frontal cortex samples for large-scale differential splicing analysis and employ PromethION sequencing to validate full-length transcript structures. Collectively, these studies demonstrate that hybrid sequencing strategies provide a practical and increasingly adopted framework for achieving both accurate quantification and comprehensive transcript structure resolution.

## Conclusion and Outlook

LrRNA-seq is fundamentally transforming transcriptomic analysis by delivering complete RNA sequences, thereby overcoming the inferential limitations of short-read technologies. Its capacity to directly resolve exon connectivity, alternative splicing events, and complex transcript architectures enables precise delineation of isoform variation and its regulatory programs. Critically, by linking specific RNA structures, such as pathogenic isoforms or unannotated transcripts, to cellular phenotypes in disease, it provides a bridge from molecular observation to functional understanding, directly informing biomarker and therapeutic development.

Future progress will require a concerted effort across 2 dimensions: (a) advancing the technology itself through cost reduction, increased throughput, and improved accuracy; and (b) establishing rigorous community guidelines for experimental design, benchmarking, and validation [1]. Direct RNA sequencing, which enables simultaneous detection of RNA sequences and modifications, offers a unique opportunity to explore the crosstalk between RNA modifications and splicing regulation. For instance, Kim et al. [182] uncover associations between m6A modification and specific splicing patterns with ONT sequencing, providing new insights into how aberrant splicing in diseases interacts with RNA modifications, stability, and translation. Specific splicing-derived isoforms have also emerged as promising prognostic biomarkers [159]. Accordingly, the development of dedicated databases that integrate disease-associated splicing patterns with detailed clinical annotations, including phenotypes, subtypes, disease stages, and prognostic outcomes, will facilitate precision medicine strategies.

Furthermore, long-read multiomics integration represents a key future direction. Integration with single-cell and spatial transcriptomics enables mapping of isoform expression and processing dynamics across individual cells and tissue architectures. When combined with nascent RNA sequencing, lrRNA-seq captures transient transcriptional kinetics and cotranscriptional splicing, revealing how RNA polymerase II dynamics directly shape exon usage [183]. In addition, long-read ribosome profiling links isoform identity to translation efficiency, enabling simultaneous quantification of mRNA abundance and ribosome engagement at the isoform level under varying conditions [184]. Together, these converging methodologies establish lrRNA-seq as a central platform for multidimensional transcriptomic interrogation.
